# Longitudinal Assessment and Cost–Benefit Analysis of Prophylactic Fenestration in Chondrodystrophic Dogs With Follow‐Up Magnetic Resonance Imaging

**DOI:** 10.1111/jvim.70191

**Published:** 2025-07-18

**Authors:** Daniel Low, Vasileios Vallios, Tomas Basto, Marios Charalambous

**Affiliations:** ^1^ frank. Pet Surgeons. Leeds UK; ^2^ Swift Referrals Wetherby UK; ^3^ St George's Vets Birmingham UK; ^4^ Blaise Veterinary Referral Hospital Birmingham UK; ^5^ University of Veterinary Medicine Hannover Hannover Germany

**Keywords:** intervertebral disc extrusion, neuroimaging, neurology, neurosurgery

## Abstract

**Background:**

Prophylactic fenestration (PF) has been reported to protect against recurrent intervertebral disc extrusion (IVDE), but recurrence is not always confirmed. No published studies address the cost–benefit of PF.

**Objectives:**

Observe the association between PF and intervertebral disc (IVD) survival and conduct cost–benefit analysis.

**Animals:**

Eighty chondrodystrophic dogs with recurrent IVDE.

**Methods:**

Longitudinal assessment with follow‐up magnetic resonance imaging (MRI) was performed to retrospectively observe the survival of in situ IVDs. The association between PF and IVD survival was analyzed using a multivariable survival model, which included Pfirrmann grade as a covariate. Worst‐case, base‐case, and best‐case cost–benefit simulations were conducted, corresponding to the lower 95% confidence interval (CI), point estimate, and upper 95% CI of the effect size of PF.

**Results:**

For IVDs treated with PF, 4/31 (12.9%) IVDs were documented to subsequently extrude on follow‐up MRI. For IVDs not treated with PF, 76/602 (12.6%) were documented to subsequently extrude on follow‐up MRI. There was no association between the use of PF and IVD survival (time ratio: 1.17; 95% CI: 0.49–2.76; *p* = 0.72). An increase in Pfirrmann grade was associated with a decrease in IVD survival (time ratio: 0.34; 95% CI: 0.26–0.46; *p* < 0.001). Prophylactic fenestration was only cost‐effective under a limited range of conditions.

**Conclusions and Clinical Importance:**

There was no evidence that PF prevented IVDE under study conditions. Pfirrmann grade was the strongest predictor of IVD survival. Universal use of PF was not cost‐effective in multiple simulations. Targeted PF of high‐risk IVDs may be considered the most cost‐effective approach.

**Abbreviations**
AFT: accelerated failure time
CI: confidence interval
IQR: interquartile range
IVD: intervertebral disc
IVDE: intervertebral disc extrusion
MRI: magnetic resonance imaging
PF: prophylactic fenestration
PH: proportional hazards

## 1 Introduction

Thoracolumbar intervertebral disc extrusion (IVDE) is a leading cause of pelvic limb neurologic dysfunction in dogs [1, 2]. The condition is especially prevalent in chondrodystrophic breeds, with certain varieties of Dachshund showing prevalence rates as high as 24.4% [3]. The thoracolumbar IVDs are overrepresented when considering first‐time and subsequent IVDE, but caudal lumbar IVDs also can be affected by the same disease process [1, 4, 5]. Chondroid degeneration of the nucleus pulposus is associated with the FGF4 retrogene [6]. Despite successful management of the first episode of IVDE, the genetic factors driving IVD degeneration remain unchanged, with clinical recurrence rates of up to 19.2% having been reported [7].

Fenestration of IVDs can be performed at the time of surgical decompression, and can be therapeutic [8, 9] or prophylactic [1]. When performed at the site of extrusion, fenestration aims to prevent further extrusion of an already ruptured IVD, which is the principal cause of early recurrence within 28 days after decompressive surgery [10, 11]. Prophylactic fenestration (PF) may be performed at non‐extruded IVD spaces to decrease the risk of recurrent IVDE [12, 13]. However, the evidence supporting the efficacy of PF is of low quality [1, 14]. Previous studies investigating the effect of PF on recurrent IVDE, including a prospective randomized controlled trial [7, 12, 13], were limited by the absence of follow‐up imaging in all dogs and dependence on follow‐up by contact with the referring veterinarian or client [14]. These methods often lead to both under‐ and over‐estimation of recurrent IVDE rates and introduce uncontrolled bidirectional bias into the results. Moreover, when follow‐up is based on the neurologic examination findings and without follow‐up imaging, the impact of PF on individual IVDs remains unclear.

Regarding decision‐making for prophylaxis of recurrent IVDE, the cost–benefit ratio of PF remains undefined [1]. Prophylactic fenestration requires additional surgical and anesthetic time, as well as further tissue dissection, all of which contribute to both surgical morbidity and the overall cost of veterinary care. Furthermore, PF is associated with adverse events such as hemorrhage, vertebral instability, further extrusion, iatrogenic discospondylitis, and nerve root trauma, but these are rare when performed by experienced surgeons, with a published complication rate of 0.01% [1, 13, 15, 16, 17].

Given the limitations of previous studies and the uncertain cost–benefit ratio of PF as an adjunctive procedure during decompressive surgery for IVDE, we aimed to evaluate the effect of PF on IVD survival with follow‐up imaging, and to conduct a cost–benefit analysis. The null hypothesis tested was that there is no association between PF and the survival of non‐extruded IVDs.

## 2 Methods

### 2.1 Study Design and Sample Population

Our study was reported in accordance with the STROBE‐VET statement [18]. All data in the study was obtained retrospectively with written client consent granting permission for the anonymized use of patient data for research purposes. A retrospective observational cohort study was conducted using a medical record search (2019–2025) of the neurology caseload of two veterinary referral hospitals in the United Kingdom. In our study, acute IVDE was defined as clinical signs of ≤ 10 days, and restricted to the IVDs between T11–T12 and L6–L7, inclusive. First IVDE was defined as the first episode of acute IVDE investigated and treated at the referral hospital. Recurrent IVDE was defined as a second IVDE episode at least 28 days after the first IVDE episode, at a distant site from the first, and also documented and treated at the same referral hospital. Chondrodystrophic dogs were defined as those from a defined range of pure breeds. In crossbred dogs, chondrodystrophic conformation and age of onset of first IVDE were used to define them as chondrodystrophic [19]. Inclusion criteria were (1) chondrodystrophic dogs with recurrent IVDE, (2) if both episodes were acute in onset, (3) and if MRI documentation of each episode was available. The diagnosis of IVDE was made based on MRI [20] and confirmed intraoperatively if decompressive surgery was carried out. Dogs were excluded if (1) they had concurrent clinically relevant myelopathies other than IVDE, (2) they experienced recurrent IVDE within 28 days, (3) contemporaneous MRI documentation of either episode was not available, (4) the extruded IVD could not be identified, or (5) the outcome from the management of the first IVDE episode was unsuccessful. A successful outcome was defined as complete resolution of all neurologic signs which, in non‐ambulatory cases, was recovery of ambulation. For dogs exhibiting only spinal pain, success was determined by resolution of vertebral column hyperesthesia. In dogs with ambulatory paraparesis, improvement in the degree of neurological deficits was considered a successful outcome.

Dogs underwent MRI of the thoracolumbar spine under general anesthesia, which was not standardized in our multicenter retrospective study. Imaging was performed with a Magnetom Aera 1.5T (Siemens AG; Germany) or Signa Explorer 1.5T (GE Healthcare; IL, USA) unit with patients in dorsal recumbency. Routine T2‐weighted sagittal and transverse sequences were obtained, with additional sequences obtained at the discretion of the attending clinician. In dogs where decompressive surgery was carried out, the thoracolumbar spine was approached dorsally for standard hemilaminectomy or pediculectomy. Fenestration was performed at the discretion of the attending clinician.

### 2.2 Data and Outcome Collection

Clinical data collected included age, breed, sex, and neuter status, body weight, neurologic examination findings, duration of clinical signs, sites of extrusion, whether surgical or non‐surgical management was performed, Pfirrmann grade [21] of IVDs within the T11‐L7 anatomical region, attending clinician, fenestration data, and interval between MRI scans. Pfirrmann grade 1 IVDs were normal in shape and signal intensity. Pfirrmann grade 2–4 discs had a progressive loss of T2‐weighted signal of the nucleus pulposus and definition between the nucleus and the annulus. Pfirrmann grade 5 discs showed collapse of the disc space. All in situ IVDs within the T11‐L7 region were entered in the study. The extruded IVD at the time of first IVDE was censored and not eligible for study inclusion, whether the IVD was fenestrated or not. Fenestration data were collected for each of the IVDs included in the study. Fenestration variables collected included the binary fenestration status of the IVD, the method of fenestration, whether the IVD was adjacent to a fenestrated IVD, whether the IVD was adjacent to an extruded IVD, and adverse events associated with fenestration. Intervertebral disc survival was the primary outcome evaluated in our study and was defined binarily. Intervertebral disc survival was determined on follow‐up MRI using the same diagnostic criteria. Intervertebral discs were excluded if they were not within the field‐of‐view on either MRI scan because their survival status could not be confirmed.

### 2.3 Statistical and Cost–Benefit Analysis

Exploratory univariable analysis was performed to assess the association between IVD survival and age, breed, sex and neuter status, body weight, site of extrusion, Pfirrmann grade, and fenestration variables. Continuous variables were compared using the Kruskal–Wallis test or one‐way analysis of variance (ANOVA) for nonparametric and parametrically distributed variables, respectively. Categorical variables were compared using the chi‐squared test or Fisher's exact test if cell values were fewer than five. To determine the appropriate survival model, variables were tested for compliance with the proportional hazards (PH) assumption. A Cox PH model was used if the assumption was met; otherwise, an accelerated failure time (AFT) model was applied. A random effects model was used to assess potential clustering by individual dogs and by institution. Multivariable analysis was conducted to model the relationship between fenestration and IVD survival, with covariate selection guided by the Akaike information criterion. Fenestration, as the primary intervention of interest, was retained in the final model regardless of statistical significance. The possibility of a variable effect of fenestration across various Pfirrmann grades and anatomical locations was tested by interaction terms introduced into multivariable analysis during model building. The association between fenestration and included covariates on IVD survival was reported as the exponentiated coefficient output of the AFT model—the time ratio (TR), with a 95% confidence interval (CI). Positive TRs were associated with increased survival, whereas negative TRs were associated with decreased survival of the IVD. Raw IVD survival was visualized using Kaplan–Meier survival curves, whereas covariate‐adjusted survival was modeled using Cox PH or AFT survival curves, depending on whether the PH assumption was met. Model significance was assessed by comparing the full model to the null model using the chi‐squared test.

The cost of decompressive surgery was estimated by a web search of publicly available fixed‐price fees for decompressive thoracolumbar hemilaminectomy in the United Kingdom, and a mean value was used as the estimate. Surgical time was estimated to be 90 min, with the surgical fee comprising 40% of the mean total price. A per‐minute surgical fee was derived accordingly. Fenestration was estimated to require 5 min per site, allowing for an estimation of the direct cost of PF. Baseline IVD survival time without fenestration was estimated empirically from the dataset (Supporting Information). The increase or decrease in IVD survival for fenestration was obtained by exponentiating the coefficient from the appropriate survival model. Adjusted survival times with fenestration were calculated for three scenarios—worst‐case, base‐case, and best‐case, each corresponding to the lower 95% CI, point estimate, and upper 95% CI of the TR, respectively. The adjusted survival times were used to estimate the probability of at least one future extrusion across seven PF scenarios in the high‐risk thoracolumbar region of T11‐L4 [1], ranging from no fenestration to fenestration of all six sites. The projected costs were estimated by multiplying the probability of extrusion by the full cost of decompressive thoracolumbar hemilaminectomy. Net cost savings were calculated by subtracting the projected costs and the direct cost of fenestration from the baseline cost (i.e., the cost associated with not performing fenestration). Statistically significant covariates from multivariable analysis were included in the cost–benefit analysis to simulate additional scenarios in addition to the baseline simulation.

Data normality was assessed using the Shapiro–Wilk test. Parametric data were reported as mean and SD. Nonparametric data were reported as median and interquartile range (IQR). Statistical significance was defined as *p* < 0.05. Statistical analysis and data visualization were performed with *survival*, *survminer*, *readr*, *ggplot2*, *dplyr*, and *tidyr* in R version 4.4.3 [22, 23, 24, 25, 26, 27].

## 3 Results

The medical record search yielded 107 consecutive dogs with recurrent IVDE and contemporaneous MRI documentation of each episode. Nine dogs were excluded because of concurrent myelopathies (intervertebral disc protrusion, *n* = 6; clinically relevant vertebral malformations, *n* = 3). Seven dogs were excluded for having non‐acute IVDE. Six dogs were excluded for recurrent IVDE within 28 days; all recurrences were at the site of initial extrusion. Four dogs were excluded for having dispersive disc extrusions where the extruded disc could not be identified. One dog was presented with paraplegia without deep pain perception and was excluded because of an unsuccessful outcome after decompressive surgery at the time of the first IVDE, despite documentation of recurrent IVDE. In total, 80 dogs met inclusion criteria for the study. Summary statistics of the sample population are provided in Table 1. Seventy‐seven of 80 (96.3%) dogs underwent surgical decompression for the first IVDE episode. All 77 dogs experienced a successful outcome, as pre‐defined by study inclusion criteria. The remaining three dogs (all ambulatory paraparetic) were managed non‐surgically with success. Of the dogs receiving decompressive surgery, the attending clinician was a board‐certified neurologist (European College of Veterinary Neurology or American College of Veterinary Internal Medicine) in 42/77 cases (54.5%), a board‐certified surgeon (European College of Veterinary Surgeons) in 18/77 cases (23.4%), or a non‐board‐certified clinician with neurosurgical experience in 20/77 cases (26.0%).

**TABLE 1:** Summary statistics of the sample population.

		*n* (%)	Median (IQR)
Demographic data			
Breed	Dachshund	50 (62.5%)	
	Crossbreed	12 (15.0%)	
	French Bulldog	9 (11.3%)	
	Other	9 (11.3%)	
Sex	Male entire	13 (16.3%)	
	Male neutered	34 (42.5%)	
	Female entire	10 (12.5%)	
	Female neutered	23 (28.8%)	
Bodyweight			7.3 kg (5.9–9.3)
First IVDE episode			
Age			4.8 years (3.8–5.3)
Duration of onset			2 days (1–4)
Neurological examination	Spinal hyperaesthesia only	2 (2.5%)	
	Ambulatory paraparesis	26 (32.5%)	
	Non‐ambulatory paraparesis	30 (37.5%)	
	Paraplegic with intact deep pain perception	17 (21.3%)	
	Paraplegic without deep pain perception	5 (6.3%)	
Second IVDE episode			
Age			5.4 years (4.6–6.8 years)
Duration of onset			2 days (1–4)
Neurological examination	Spinal hyperaesthesia only	6 (7.5%)	
	Ambulatory paraparesis	30 (37.5%)	
	Non‐ambulatory paraparesis	32 (40.0%)	
	Paraplegic with intact deep pain perception	8 (10.0%)	
	Paraplegic without deep pain perception	4 (5.0%)	

Abbreviations: IQR, interquartile range; IVDE, intervertebral disc extrusion.

Single‐site fenestration was performed in 20/77 dogs (26.0%), multi‐level fenestration was performed in 22/77 dogs (28.6%), and no fenestration was performed in the remaining 35/77 dogs (45.5%). All fenestration procedures were performed using the blade technique. The use of fenestration was significantly different (*p* < 0.001) among board‐certified neurologists (single site, *n* = 10; multi‐level, *n* = 21; none, *n* = 11), board‐certified surgeons (single site, *n* = 9; multi‐level, *n* = 1; none, *n* = 8), and non‐board‐certified clinicians (single site, *n* = 1; none, *n* = 0). Eighty‐seven IVDs were excluded because of not being within the field‐of‐view. A total of 633 in situ IVDs within the T11‐L7 region were included in the study, and 31 (4.9%) of these received PF. No significant difference was found in the location of PF across the T11‐L7 region (*p* = 0.07).

No adverse events were recorded in association with PF. Two‐hundred and fifteen IVDs (34.0%) were Pfirrmann grade 2, 258 IVDs (40.8%) were Pfirrmann grade 3, and 160 IVDs (25.3%) were Pfirrmann grade 4. At the IVD level, the median interval between first and recurrent IVDE episodes was 231 days (IQR: 116–417 days). All 80 dogs in the sample population experienced recurrent IVDE, comprising a total of 80 IVDs documented not to survive, as pre‐defined by study inclusion criteria. Of the 31 IVDs that received PF, four IVDs (12.9%) subsequently were documented to extrude (L1–L2, *n* = 2; L2–L3, *n* = 2). Of the 602 IVDs that did not undergo PF, 76 (12.6%) subsequently were documented to extrude on follow‐up MRI. None of the recurrent extrusions were at the same site as the first IVDE. Pfirrmann grade was significantly associated with IVD survival on univariable analysis (*p* < 0.001). Fenestration status was not associated with IVD survival on univariable analysis, nor were any of the other variables tested. The PH assumption was violated (*p* = 0.049) and therefore an AFT model was used. Neither within‐dog clustering (*p* = 0.70) nor institutional clustering (*p* = 0.88) was observed on the random effects model. No significant interactions were observed between PF and Pfirrmann grade (*p* = 0.40) and PF and anatomical location of the IVD (*p* = 0.60). The final multivariable AFT model modeled the association between PF and IVD survival, with Pfirrmann grade, being adjacent to a fenestrated IVD, and being adjacent to an extruded IVD as covariates. Compared to the null model, the full model was significant with a chi‐squared statistic of 87.73 on four degrees of freedom (*p* < 0.001).

**FIGURE 1:**
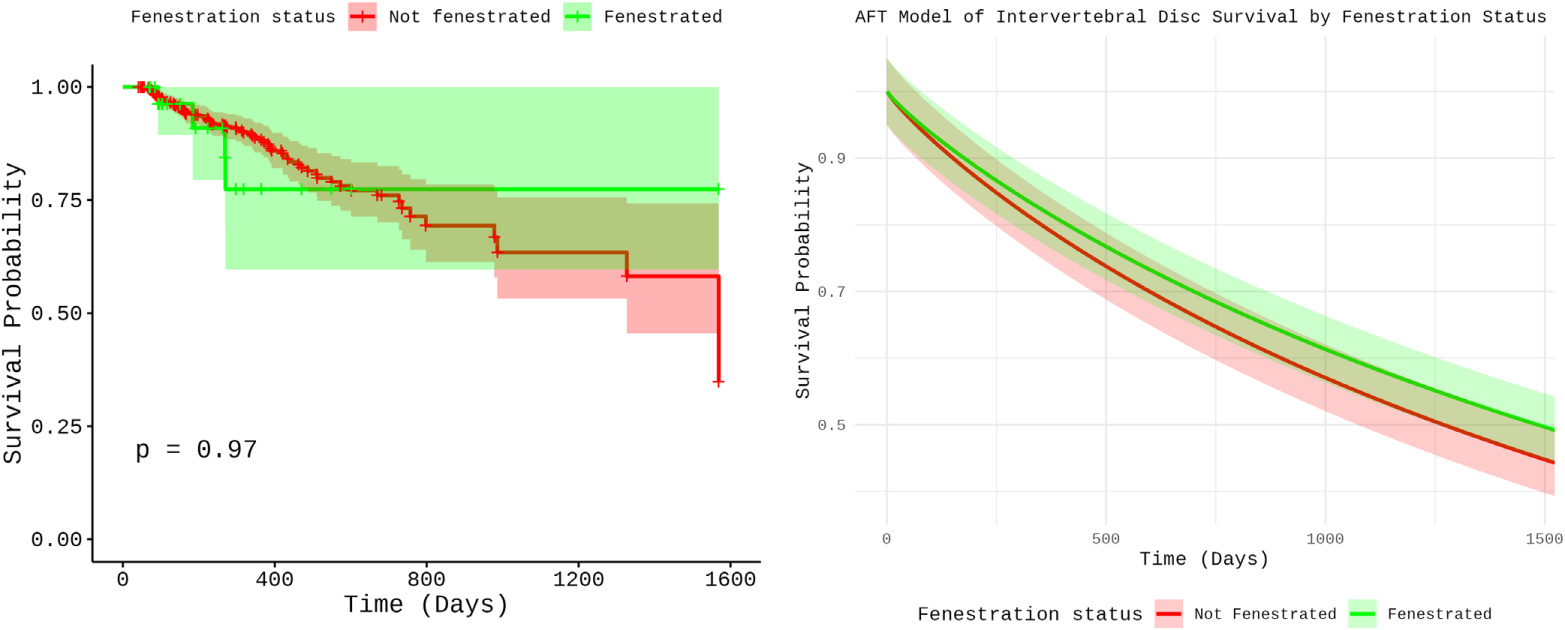
Kaplan–Meier survival curve (left) depicting the unadjusted survival probability of fenestrated and non‐fenestrated intervertebral discs over time. Accelerated Failure Time (AFT) model‐derived survival curve (right) illustrating the adjusted survival probabilities after accounting for covariates in the multivariate analysis.

**FIGURE 2:**
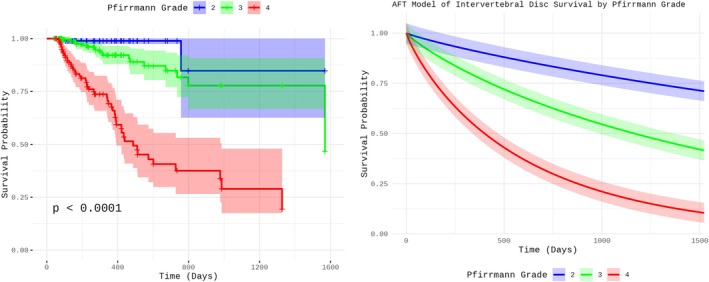
Kaplan–Meier survival curve (left) depicting the unadjusted survival probability of intervertebral discs by Pfirrman grade over time. Accelerated Failure Time (AFT) model‐derived survival curve (right) illustrating the adjusted survival probabilities after accounting for covariates in the multivariate analysis.

On multivariable analysis, PF was not associated (*p* = 0.72) with extended or decreased IVD survival (Figure 1), with a TR of 1.17 (95% CI: 0.49–2.76). An increase of one unit in the Pfirrmann grade was associated (*p* < 0.001) with a decrease in IVD survival (Figure 2), with a TR of 0.34 (95% CI: 0.26–0.46). Being adjacent to a fenestrated IVD was not associated (*p* = 0.44) with IVD survival, with a TR of 0.82 (95% CI: 0.49–1.37). Being adjacent to an extruded IVD was not associated (*p* = 0.13) with IVD survival, with a TR of 0.73 (95% CI: 0.48–1.10).

**FIGURE 3:**
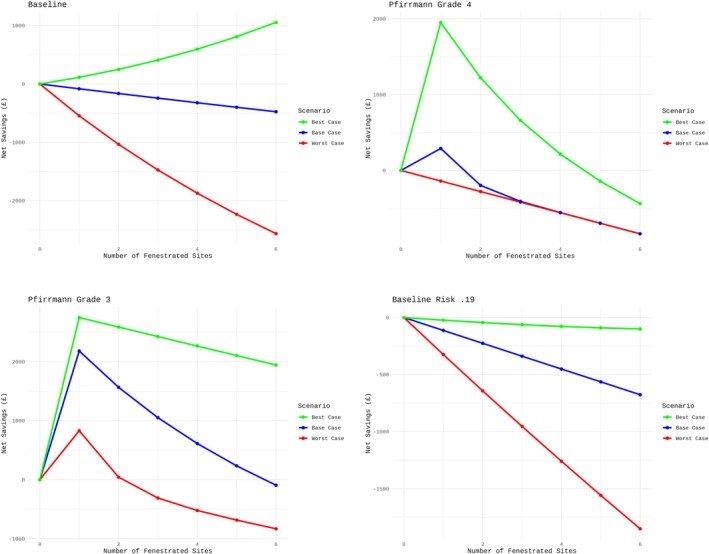
Cost–benefit analysis in the sample population (top‐left), in Pfirrmann grade 4 discs (top‐right), in Pfirrmann grade 3 discs (bottom‐left), and in an external population (bottom‐right), estimating the net savings associated with different fenestration strategies across worst‐case (red line), base‐case (blue line), and best‐case (green line) scenarios corresponding to the lower 95% CI, point estimate, and upper 95% CI of the effect size of prophylactic fenestration.

Fixed‐price fees of decompressive thoracolumbar hemilaminectomy were obtained from 17 referral centers, with a mean of £6267.06 (SD = 1185.45; range: £3995–£8000). Fenestration of one IVD space was estimated to cost £139.25. Baseline survival time of non‐fenestrated IVDs in our study was 231 days. As a baseline, the probability of at least one IVD extrusion in this sample population was 0.5356 without PF. Cost–benefit analysis was simulated for baseline risk of the sample population, on Pfirrmann grade 4 discs, on Pfirrmann grade 3 discs, and on an external population with a baseline risk of 0.19 (Figure 3). Simulation of the baseline cost–benefit of PF showed no net savings from PF in the worst‐case and base‐case scenarios. In the best‐case scenario, PF showed increasing net savings as the number of IVDs treated increased. In the simulation of PF on Pfirrmann grade 4 discs, PF resulted in net savings when four or fewer IVDs were treated in the best‐case scenario. In the base‐case scenario, PF resulted in net savings only when treating one Pfirrmann grade 4 IVD with PF. In the worst‐case scenario, PF did not result in net savings when treating Pfirrmann grade 4 IVDs. In the simulation of PF on Pfirrmann grade 3 discs, PF resulted in net savings under all conditions in the best‐case scenario. In the base‐case scenario, PF resulted in net savings when five or fewer Pfirrmann grade 3 IVDs were treated with PF. In the worst‐case scenario, PF resulted in net savings when one Pfirrmann grade 3 IVD was treated, was almost cost‐neutral when two Pfirrmann grade 3 IVDs were treated, and resulted in no net savings in the remaining conditions. Simulation of PF on an external population showed that PF was not cost‐effective in any of the conditions tested. Cost–benefit analysis formulae and raw calculations are provided in Supporting Information.

## 4 Discussion

We did not identify a protective effect of PF on the in situ IVD and therefore insufficient evidence was found to reject the null hypothesis. We demonstrated that an increase in Pfirrmann grade was associated with a decrease in IVD survival. We present cost–benefit analyses across various simulations to demonstrate the conditions under which PF may be considered cost‐effective.

The protective effects of PF previously have been questioned, with previous studies reporting beneficial effects of PF limited by the nature of outcome data collection [14]. These studies [7, 12, 13, 28] did not systematically use follow‐up imaging or surgery to confirm recurrent IVDE and, instead, relied on a combination of methods to identify recurrent IVDE. When relying on telephone follow‐up, clinical signs may be misinterpreted or missed by dog owners, leading to false positives and false negatives, respectively. The neurologic examination is more reliable than telephone follow‐up, and although it may allow a clinical diagnosis of recurrent IVDE, it cannot provide definitive confirmation of the diagnosis or site of extrusion without imaging. Clinical reasoning without systematic diagnostic imaging is widely accepted in veterinary practice [29], but research settings require precise case classification to minimize the risk of bias and ensure valid study outcomes. Loss to follow‐up, regardless of follow‐up methodology, also biases results in that non‐followed‐up cases are classified as non‐recurrent. However, multiple scenarios exist in which a recurrent IVDE case remains unknown to the referral hospital for reasons beyond the referral hospital's control [30]. These include, but are not limited to, non‐recognition of clinical signs, the referring veterinarian managing the case, treatment at another referral hospital, or euthanasia because of recurrent IVDE. In our study, these biases were mitigated with all included dogs receiving follow‐up neurologic examination and follow‐up imaging.

The main finding of our study was that PF was not associated with IVD survival. In general, biomedical research with a higher risk of bias tends toward reporting larger effect sizes associated with an intervention [31, 32]. Our study accounted for the aforementioned biases in that we precisely identified the effect of PF on IVDs within the thoracolumbar region. Our findings disagree with prior studies, which may reflect misclassification of recurrent and non‐recurrent IVDE cases and therefore an exaggerated protective effect of PF in previous studies. The wide CIs associated with the point estimate of the survival‐modifying effect of PF reflect a limitation of our study, in that only a minority of IVDs received PF. Our findings do not suggest that PF is ineffective or harmful, but instead suggest that further investigation with follow‐up imaging is required to determine the true effect of PF, which may decrease, increase, or have no effect on the extrusion risk of the in situ IVD. The PH assumption was violated in our study and therefore an AFT model was used. Although less commonly used compared with Cox PH, an AFT model may be better suited to model biological systems. Cox PH analyses assume that the effect of an intervention is constant over time [33]. Biological systems do not always satisfy the assumption of PH [34, 35], and AFT models have been shown to be credible alternatives [36]. We replicate previous findings in that IVD degeneration is associated with IVD survival [37]. Pfirrmann grade has been shown to reliably assess IVD degeneration [21], and our findings suggest that IVDs with more advanced chondroid degeneration are at higher risk of non‐survival under the conditions of our study. Prophylactic fenestration previously has been suggested to simultaneously decrease the risk of extrusion at the site of fenestration and increase the risk of adjacent‐site extrusion [1, 14], however, no evidence was found in our study that fenestration or natural extrusion affected adjacent IVD survival within the studied thoracolumbar region.

We performed cost–benefit analysis to evaluate the clinical utility of PF as an intervention, which is arguably a more relevant measure than the effect size. Cost–benefit analysis is relevant in spinal surgery in humans, where the costs of an intervention are critically assessed against financial and non‐financial benefits [38]. In veterinary soft tissue surgery, prophylactic gastropexy has been shown to be effective at decreasing the risk of gastric dilatation‐volvulus but is not cost‐effective in low‐ to medium‐risk breeds [39]. Prophylactic fenestration typically is performed alongside therapeutic decompressive surgery and is associated with additional tissue dissection, increased surgical time, and prolonged anesthesia. Given that the majority of dogs do not experience recurrent IVDE even in the absence of PF [7], a quantitative cost–benefit analysis is essential to weigh the cost and benefits of PF against the costs of not performing PF. Baseline cost–benefit analysis showed that universal PF from T11 to L4 is unlikely to yield net savings, except in the best‐case scenario where a strong protective effect of PF is assumed. When cost–benefit analysis was simulated with targeted PF of Pfirrmann grade 4 discs only, PF appeared cost‐effective under best‐case conditions only, but only up to a certain point. Diminishing net savings was demonstrated as the number of Pfirrmann grade 4 discs increased, suggesting that even if PF provided substantial protection, this protection did not outweigh the cumulative costs of additional surgical procedures in high‐risk Pfirrmann grade 4 IVDs. When cost–benefit analysis was simulated with targeted PF of Pfirrmann grade 3 discs only, PF was generally cost‐effective in both the base‐ and best‐case scenarios. Targeted PF of Pfirrmann grade 3 discs was cost‐effective under a wider range of conditions, and this procedure may be considered a viable and cost‐effective approach to PF. The dogs in our study were also quantitatively demonstrated to have a high risk of recurrent IVDE with a baseline risk of 0.53, far exceeding the general population of dogs at risk of recurrent IVDE. Cost–benefit analysis in a simulated external population of dogs with a baseline risk of 0.19 showed that PF was not cost‐effective under any condition.

The cost–benefit analysis in our study represents the first analysis of the costs and benefits of PF, and the results should be interpreted with caution. The costs of recurrent IVDE and of fenestration were estimated based on fixed‐price fees for decompressive thoracolumbar hemilaminectomy packages publicly available online. Although this analysis reflects an average price across the United Kingdom, it does not account for regional variations and for unrepresented regions where prices are not published online. Our cost–benefit analysis assumes a linear increase in direct costs of PF, which may not be empirically true. Extensive PF, if extending beyond the site of decompression, requires additional dissection and may add substantially to surgical time in a non‐linear fashion, and was not accounted for in our analysis. Additionally, if PF is performed adjacent to the site of decompression, it is associated with minimal additional dissection beyond that of standard hemilaminectomy or pediculectomy. For simplicity, our analysis assumed that all cases of recurrent IVDE were managed with decompressive surgery, which does not reflect real‐world clinical decision‐making. Our data did not demonstrate an increased risk of extrusion if adjacent IVDs were fenestrated or extruded, and this assumption of independent extrusion risk was carried over into cost–benefit analysis. Our analysis only accounted for surgical time and did not account for the cost of surgical and anesthetic consumables. We did not account for less tangible costs such as surgical morbidity or the animal welfare implications of recurrent IVDE leading to pain and neurologic dysfunction. Finally, cost–benefit analysis did not account for adverse events associated with fenestration. Although adverse events were not recorded in our study and are generally extremely rare [1], these are relevant considerations for future analyses.

Our study was not designed to investigate the effect of fenestration of an already extruded disc at the time of surgery, which has been reported to be protective against early recurrence [10, 11]. Nonetheless, our findings and exclusions are in agreement with previous research in that early recurrences were exclusively at the same site, whereas late recurrences were exclusively at distant sites. Our sample population is otherwise generally representative of the population of chondrodystrophic dogs presenting with acute IVDE, with young male dogs overrepresented. Dachshunds have been reported to be at increased risk of recurrent IVDE [7, 12] and were markedly overrepresented in our sample population. Caudal lumbar IVDs were included in our analysis because doing so allowed modeling of IVD survival beyond the limited thoracolumbar region. Although there may have been potential confounding of results by breed [4, 40] and differential application of fenestration in the caudal lumbar region [1], no significant confounding was detected using the methodology in our study.

Our study is limited by its retrospective nature and uncontrolled use of PF as an intervention. We replicate practice pattern differences where board‐certified neurologists were more likely to use PF compared with board‐certified surgeons [41]. Although all fenestration procedures were performed using the same blade technique, there may have been uncontrolled differences in the quality of fenestration among attending clinicians [1]. Nonetheless, the majority (41/42) of fenestration procedures in our study were performed by board‐certified specialists, and therefore the risk of bias was minimized. The use of blade fenestration likely reflects institutional equipment availability and surgeon preference, and the results of our study may not necessarily apply to other methods of fenestration [42]. Another limitation of our study was that the imaging protocol was non‐standardized, with various additional sequences requested at the attending clinician's discretion. However, this factor unlikely affected results because standard T2‐weighted sequences are sufficient to diagnose IVDE [1], and qualitative information provided by additional non‐routine sequences was beyond the scope of our study. Specific to our study, all included cases experienced recurrent IVDE, by definition, and the results should be extrapolated with caution to the general population of dogs at risk of recurrent IVDE. Recurrence has been reported in up to 19.2% of dogs experiencing a first‐time IVDE episode [7], meaning that four‐fifths of dogs never experience clinically relevant recurrent IVDE despite not receiving PF. In cost–benefit analysis, the baseline risk of at least one future IVD extrusion without PF of the IVDs studied was empirically estimated to be 53.6%, far exceeding previous reports. The dogs included in our study likely belonged to a specific subgroup of high‐risk patients, and the effect of PF may be different when studied in a broader population. Doing so is unlikely to be possible outside of a prospective study, where follow‐up imaging in asymptomatic dogs is required to truly determine the effect of PF. Such a study would be logistically and ethically challenging to conduct [43]. Our findings also only pertain to the first and second episodes of IVDE. A small subset of dogs experience three or more episodes of recurrent IVDE and likely constitute a specific ultra‐high‐risk subset of the canine population. How our findings apply to these dogs and how they apply to recurrent IVDE beyond the second episode remains unknown. Finally, paraplegic dogs without deep pain perception were under‐represented in our study. Loss of deep pain perception is associated with a significantly poorer rate of recovery of ambulation and a reasonable risk of progressive myelomalacia [28, 44]. These dogs are less likely to survive or experience a functional recovery and therefore less likely to experience recurrent IVDE. As mentioned earlier, our study's results should not be used to recommend against PF, but instead should prompt further research with follow‐up imaging to determine the true effect of PF, protective or otherwise.

In conclusion, our study did not show a protective effect of PF on recurrent IVDE. We showed that Pfirrmann grade was the strongest predictor of IVD survival. Universal PF is unlikely to be cost‐effective, both in high‐risk dogs and in the general population of dogs susceptible to recurrent IVDE. Targeted PF was cost‐effective, under certain conditions only. Further research incorporating follow‐up imaging is essential to thoroughly understand the true effect of PF on recurrent IVDE and to refine the cost–benefit of this procedure.

## Disclosure

Authors declare no off‐label use of antimicrobials.

## Ethics Statement

Authors declare no institutional animal care and use committee or other approval was needed. Authors declare human ethics approval was not needed.

## Conflicts of Interest

The authors declare no conflicts of interest.

## Supporting information


Data S1.

## References

[1] N. J. Olby , S. A. Moore , B. Brisson , et al., “ACVIM Consensus Statement on Diagnosis and Management of Acute Canine Thoracolumbar Intervertebral Disc Extrusion,” Journal of Veterinary Internal Medicine 36, no. 5 (2022): 1570–1596, 10.1111/jvim.16480. 35880267 PMC9511077

[2] G. Rossi , A. Stachel , A. M. Lynch , and N. J. Olby , “Intervertebral Disc Disease and Aortic Thromboembolism Are the Most Common Causes of Acute Paralysis in Dogs and Cats Presenting to an Emergency Clinic,” Veterinary Record 187, no. 10 (2020): e81, 10.1136/vr.105844. 32471959

[3] R. M. A. Packer , I. J. Seath , D. G. O'Neill , S. De Decker , and H. A. Volk , “DachsLife 2015: An Investigation of Lifestyle Associations With the Risk of Intervertebral Disc Disease in Dachshunds,” Canine Genetics and Epidemiology 3, no. 1 (2016): 8, 10.1186/s40575-016-0039-8. 27826450 PMC5097381

[4] T. Aikawa , M. Shibata , M. Asano , Y. Hara , M. Tagawa , and H. Orima , “A Comparison of Thoracolumbar Intervertebral Disc Extrusion in French Bulldogs and Dachshunds and Association With Congenital Vertebral Anomalies,” Veterinary Surgery 43, no. 3 (2014): 301–307, 10.1111/j.1532-950X.2014.12102.x. 24433331

[5] J. Abouzeid , N. Grapes , S. Khan , S. De Decker , and P. Freeman , “Comparison of Clinical Features of Intervertebral Disc Extrusions in English Cocker Spaniels, French Bulldogs and Dachshunds,” Animals 15, no. 4 (2025): 602, 10.3390/ani15040602. 40003082 PMC11851645

[6] E. A. Brown , P. J. Dickinson , T. Mansour , et al., “ *FGF4* Retrogene on CFA12 Is Responsible for Chondrodystrophy and Intervertebral Disc Disease in Dogs,” Proceedings of the National Academy of Sciences 114, no. 43 (2017): 11476–11481, 10.1073/pnas.1709082114. PMC5664524 29073074

[7] P. D. Mayhew , R. C. McLear , L. S. Ziemer , et al., “Risk Factors for Recurrence of Clinical Signs Associated With Thoracolumbar Intervertebral Disk Herniation in Dogs: 229 Cases (1994–2000),” Journal of the American Veterinary Medical Association 225, no. 8 (2004): 1231–1236, 10.2460/javma.2004.225.1231. 15521446

[8] P. Freeman and N. D. Jeffery , “Re‐Opening the Window on Fenestration as a Treatment for Acute Thoracolumbar Intervertebral Disc Herniation in Dogs,” Journal of Small Animal Practice 58, no. 4 (2017): 199–204, 10.1111/jsap.12653. 28276121

[9] N. D. Jeffery and P. M. Freeman , “The Role of Fenestration in Management of Type I Thoracolumbar Disk Degeneration,” Veterinary Clinics of North America. Small Animal Practice 48, no. 1 (2018): 187–200, 10.1016/j.cvsm.2017.08.012. 29074336

[10] S. Dhupa , N. Glickman , and D. J. Waters , “Reoperative Neurosurgery in Dogs With Thoracolumbar Disc Disease,” Veterinary Surgery 28, no. 6 (1999): 421–428, 10.1111/j.1532-950X.1999.00421.x. 10582738

[11] F. Forterre , M. Konar , D. Spreng , A. Jaggy , and J. Lang , “Influence of Intervertebral Disc Fenestration at the Herniation Site in Association With Hemilaminectomy on Recurrence in Chondrodystrophic Dogs With Thoracolumbar Disc Disease: A Prospective MRI Study,” Veterinary Surgery 37, no. 4 (2008): 399–405, 10.1111/j.1532-950X.2008.00394.x. 18564265

[12] B. A. Brisson , S. L. Moffatt , S. L. Swayne , and J. M. Parent , “Recurrence of Thoracolumbar Intervertebral Disk Extrusion in Chondrodystrophic Dogs After Surgical Decompression With or Without Prophylactic Enestration: 265 Cases (1995–1999),” Journal of the American Veterinary Medical Association 224, no. 11 (2004): 1808–1814, 10.2460/javma.2004.224.1808. 15198267

[13] B. A. Brisson , D. L. Holmberg , J. Parent , W. C. Sears , and S. E. Wick , “Comparison of the Effect of Single‐Site and Multiple‐Site Disk Fenestration on the Rate of Recurrence of Thoracolumbar Intervertebral Disk Herniation in Dogs,” Journal of the American Veterinary Medical Association 238, no. 12 (2011): 1593–1600, 10.2460/javma.238.12.1593. 21671814

[14] A. E. Pontikaki , K. Pavlidou , Z. Polizopoulou , I. Savvas , and G. Kazakos , “Prophylactic Effect of Fenestration on the Recurrence of Thoracolumbar Intervertebral Disc Disease in Dogs,” Animals 12, no. 19 (2022): 2601, 10.3390/ani12192601. 36230341 PMC9559642

[15] T. P. Hill , A. M. Lubbe , and A. J. Guthrie , “Lumbar Spine Stability Following Hemilaminectomy, Pediculectomy, and Fenestration,” Veterinary and Comparative Orthopaedics and Traumatology 13, no. 4 (2000): 165–171, 10.1055/s-0038-1632655.

[16] G. Harris and P. Freeman , “Introduction of Disc Material Into the Vertebral Canal by Fenestration of Thoracolumbar Discs Following Decompressive Surgery,” Veterinary and Comparative Orthopaedics and Traumatology 33, no. 1 (2020): 66–70, 10.1055/s-0039-1700554. 31756749

[17] M. Schwartz , I. C. Boettcher , S. Kramer , and A. Tipold , “Two Dogs With Iatrogenic Discospondylitis Caused by Meticillin‐Resistant *Staphylococcus aureus* ,” Journal of Small Animal Practice 50, no. 4 (2009): 201–205, 10.1111/j.1748-5827.2008.00720.x. 19320814

[18] J. M. Sargeant , A. M. O'Connor , I. R. Dohoo , et al., “Methods and Processes of Developing the Strengthening the Reporting of Observational Studies in Epidemiology − Veterinary (STROBE‐Vet) Statement,” Preventive Veterinary Medicine 134 (2016): 188–196, 10.1016/j.prevetmed.2016.09.005. 27836042

[19] L. A. Smolders , N. Bergknut , G. C. M. Grinwis , et al., “Intervertebral Disc Degeneration in the Dog. Part 2: Chondrodystrophic and Non‐Chondrodystrophic Breeds,” Veterinary Journal 195, no. 3 (2013): 292–299, 10.1016/j.tvjl.2012.10.011. 23154070

[20] R. C. Da Costa , S. De Decker , M. J. Lewis , and H. Volk , “The Canine Spinal Cord Injury Consortium (CANSORT‐SCI). Diagnostic Imaging in Intervertebral Disc Disease,” Frontiers in Veterinary Science 7 (2020): 588338, 10.3389/fvets.2020.588338. 33195623 PMC7642913

[21] N. Bergknut , E. Auriemma , S. Wijsman , et al., “Evaluation of Intervertebral Disk Degeneration in Chondrodystrophic and Nonchondrodystrophic Dogs by Use of Pfirrmann Grading of Images Obtained With Low‐Field Magnetic Resonance Imaging,” American Journal of Veterinary Research 72, no. 7 (2011): 893–898, 10.2460/ajvr.72.7.893. 21728849

[22] A. Kassambara , M. Kosinski , and P. Biecek , “survminer: Drawing Survival Curves Using ‘ggplot2’,” published January 18, 2016, 10.32614/CRAN.package.survminer.

[23] T. M. Therneau , “survival: Survival Analysis,” 2001 published June 22, 10.32614/CRAN.package.survival.

[24] H. Wickham , J. Hester , and J. Bryan , “readr: Read Rectangular Text Data,” published April 9, 2015, 10.32614/CRAN.package.readr.

[25] H. Wickham , R. François , L. Henry , K. Müller , and D. Vaughan , “dplyr: A Grammar of Data Manipulation,” published January 16, 2014, 10.32614/CRAN.package.dplyr.

[26] H. Wickham , D. Vaughan , and M. Girlich , “tidyr: Tidy Messy Data,” published July 21, 2014, 10.32614/CRAN.package.tidyr.

[27] H. Wickham , Ggplot2: Elegant Graphics for Data Analysis (Springer‐Verlag New York, 2016).

[28] T. Aikawa , H. Fujita , M. Shibata , and T. Takahashi , “Recurrent Thoracolumbar Intervertebral Disc Extrusion After Hemilaminectomy and Concomitant Prophylactic Fenestration in 662 Chondrodystrophic Dogs,” Veterinary Surgery 41, no. 3 (2012): 381–390, 10.1111/j.1532-950X.2012.00970.x. 22380868

[29] T. J. A. Cardy , S. De Decker , P. J. Kenny , and H. A. Volk , “Clinical Reasoning in Canine Spinal Disease: What Combination of Clinical Information Is Useful?,” Veterinary Record 177, no. 7 (2015): 171, 10.1136/vr.102988. 26198211

[30] D. Low and S. Rutherford , “Investigating the Weekend Effect in Decompressive Thoracolumbar Hemilaminectomy for Acute Intervertebral Disc Extrusion: An Observational Cohort Study of 460 Cases (2018–2023),” Veterinary Surgery 53 (2024): vsu.14089, 10.1111/vsu.14089. 38556784

[31] J. Savović , R. M. Turner , D. Mawdsley , et al., “Association Between Risk‐Of‐Bias Assessments and Results of Randomized Trials in Cochrane Reviews: The ROBES Meta‐Epidemiologic Study,” American Journal of Epidemiology 187, no. 5 (2018): 1113–1122, 10.1093/aje/kwx344. 29126260 PMC5928453

[32] S. Schwab , G. Kreiliger , and L. Held , “Assessing Treatment Effects and Publication Bias Across Different Specialties in Medicine: A Meta‐Epidemiological Study,” BMJ Open 11, no. 9 (2021): e045942, 10.1136/bmjopen-2020-045942. PMC8442042 34521659

[33] W. R. Swindell , “Accelerated Failure Time Models Provide a Useful Statistical Framework for Aging Research,” Experimental Gerontology 44, no. 3 (2009): 190–200, 10.1016/j.exger.2008.10.005. 19007875 PMC2718836

[34] M. Parsa and I. Van Keilegom , “Accelerated Failure Time Vs Cox Proportional Hazards Mixture Cure Models: David Vs Goliath?,” Statistical Papers 64, no. 3 (2023): 835–855, 10.1007/s00362-022-01345-5.

[35] Y. A. Mustefa and D. G. Chen , “Accelerated Failure‐Time Model With Weighted Least‐Squares Estimation: Application on Survival of HIV Positives,” Archives of Public Health 79, no. 1 (2021): 88, 10.1186/s13690-021-00617-0. 34059125 PMC8165794

[36] A. Zare , M. Hosseini , M. Mahmoodi , K. Mohammad , H. Zeraati , and K. Holakouie Naieni , “A Comparison Between Accelerated Failure‐Time and Cox Proportional Hazard Models in Analyzing the Survival of Gastric Cancer Patients,” Iranian Journal of Public Health 44, no. 8 (2015): 1095–1102. 26587473 PMC4645729

[37] S. Longo , S. A. Gomes , C. Briola , et al., “Association of Magnetic Resonance Assessed Disc Degeneration and Late Clinical Recurrence in Dogs Treated Surgically for Thoracolumbar Intervertebral Disc Extrusions,” Journal of Veterinary Internal Medicine 35, no. 1 (2021): 378–387, 10.1111/jvim.15989. 33283382 PMC7848362

[38] S. L. Parker , L. H. Anderson , T. Nelson , and V. V. Patel , “Cost‐Effectiveness of Three Treatment Strategies for Lumbar Spinal Stenosis: Conservative Care, Laminectomy, and the Superion Interspinous Spacer,” International Journal of Spine Surgery 9 (2015): 28, 10.14444/2028. 26273546 PMC4528571

[39] M. P. Ward , G. J. Patronek , and L. T. Glickman , “Benefits of Prophylactic Gastropexy for Dogs at Risk of Gastric Dilatation–Volvulus,” Preventive Veterinary Medicine 60, no. 4 (2003): 319–329, 10.1016/S0167-5877(03)00142-9. 12941556

[40] T. J. A. Cardy , C. E. Tzounos , H. A. Volk , and S. De Decker , “Clinical Characterization of Thoracolumbar and Lumbar Intervertebral Disk Extrusions in English Cocker Spaniels,” Journal of the American Veterinary Medical Association 248, no. 4 (2016): 405–412, 10.2460/javma.248.4.405. 26829272

[41] S. A. Moore , P. J. Early , and B. F. Hettlich , “Practice Patterns in the Management of Acute Intervertebral Disc Herniation in Dogs,” Journal of Small Animal Practice 57, no. 8 (2016): 409–415, 10.1111/jsap.12496. 27256593

[42] D. L. Holmberg , N. C. Palmer , D. Vanpelt , and A. R. Willan , “A Comparison of Manual and Power‐Assisted Thoracolumbar Disc Fenestration in Dogs,” Veterinary Surgery 19, no. 5 (1990): 323–327, 10.1111/j.1532-950X.1990.tb01199.x. 2145687

[43] S. A. Moore , A. Tipold , N. J. Olby , V. Stein , N. Granger , and CANSORT‐SCI , “Current Approaches to the Management of Acute Thoracolumbar Disc Extrusion in Dogs,” Frontiers in Veterinary Science 7 (2020): 610, 10.3389/fvets.2020.00610. 33117847 PMC7521156

[44] N. J. Olby , R. C. da Costa , J. M. Levine , and V. M. Stein , “Prognostic Factors in Canine Acute Intervertebral Disc Disease,” Frontiers in Veterinary Science 7 (2020): 596059, 10.3389/fvets.2020.596059. 33324703 PMC7725764

