# Supplementary material for: Longitudinal Assessment and Cost–Benefit Analysis of Prophylactic Fenestration in Chondrodystrophic Dogs With Follow‐Up Magnetic Resonance Imaging
Source: J Vet Intern Med. 2025 Jul 18;39(4):e70191. doi: 10.1111/jvim.70191 (PMC12271825; doi:10.1111/jvim.70191)
Supplement: Supplementary file 1 — Data S1. [file JVIM-39-e70191-s001.pdf]

Supplementary File 1

Prices correct as of 22/03/2025

Location of referral centre	Fixed price package (£)
Godalming, Surrey	7500
Bletchingley, Surrey	7350
London	7500
Dorset	5000
Northamptonshire	3995
Wiltshire	6500
Bristol	5500
Cambridgeshire	5995
Birmingham	5750
Wetherby, West Yorkshire	7900
Wakefield, West Yorkshire	8000
Leeds	5000
Cheshire	7550
Cumbria	5000
Northumberland	5800
County Durham	6850
Edinburgh	5350
**Mean value**	**6267.06**

$$Estimate\ of\ decompressive\ surgery\ fees\ =\ 40\%\ \times\ £6267.06 = £2506.82$$

$$Estimate\ of\ surgical\ fees\ per\ minute = \frac{2506.82}{90} = £27.85$$

$$Estimate\ of\ direct\ cost\ of\ one\ site\ fenestration = 5\ \times\ 27.85 = £139.25$$

$$Baseline\ disc\ survival\ time\ (BST)\ without\ fenestration\ =\ 231\ days$$

$$Time\ ratio\ (TR)\ =\ e^{\ coefficient}$$

$$Adjusted\ disc\ survival\ time\ (AST)\ with\ fenestration\ =\ 231\ \times\ TR$$

$$TR_{\ WorstCase} = 0.4947;\ TR_{\ BaseCase} = 1.169;\ TR_{\ BestCase} = 2.762$$

Baseline cost benefit analysis

$$AST_{\ WorstCase} = 231\ \times\ 0.4947\ =\ 114.28$$

$$AST_{\ BaseCase} = 231\ \times\ 1.169\ =\ 270.04$$

$$AST_{\ BestCase} = 231\ \times\ 2.762\ =\ 638.02$$

$$P_{\ fen\ extrusion} = P_{\ non-fen\ extrusion} \times \frac{BST}{AST}$$

$$P_{\ non-fen\ extrusion} = 0.12$$

$$P_{\ fen\ extrusion,\ worst\ case} = 0.12\ \times \frac{231}{114.28} = 0.2426$$

$$P_{\ fen\ extrusion,\ base\ case} = 0.12\ \times \frac{231}{270.04} = 0.1027$$

$$P_{\ fen\ extrusion,\ best\ case} = 0.12\ \times \frac{231}{638.02} = 0.04345$$

Consider prophylactic fenestration of 0-6 sites between T11-L4 (T11-T12, T12-T13, T13-L1, L1-L2, L2-L3, L3-L4).

$$P_{\ 1\leq\ extrusion} = 1\ -\ (1\ -\ P_{\ fen\ extrusion,\ X\ case})^{\ \#\ fenestrated\ discs} \times\ (1\ -\ P_{\ non-fen\ extrusion})^{\ 6-\#\ fenestrated\ discs}$$

$P_{1\leq extrusion}$ of various cases amongst 0-6 site fenestration				
Sites fenestrated	Worst case	Base case	Best case	Direct cost (£)

0 sites	0.5356	0.5356	0.5356	0.00
1 site	0.6003	0.5265	0.4952	139.25
2 sites	0.6560	0.5172	0.4513	278.50
3 sites	0.7039	0.5076	0.4036	417.75
4 sites	0.7451	0.4980	0.3517	557.00
5 sites	0.7807	0.4881	0.2953	696.25
6 sites	0.8112	0.4781	0.2340	835.50

$$Projected\ costs\ =\ P_{1\leq\ extrusion} \times \text{£}6267.06$$

$$Total\ costs\ =\ Projected\ costs\ +\ direct\ cost\ of\ fenestration$$

$$Net\ savings\ =\ 0\ site\ fenestration\ costs\ -\ total\ costs$$

Baseline cost-benefit analysis							
Sites fenestrated	Projected costs worst case	Projected costs base case	Projected costs best case	Direct cost	Net savings worst case	Net savings base case	Net savings best case
0 sites	3356.64	3356.64	3356.64	0.00	0.00	0.00	0.00
1 site	3762.12	3299.61	3103.45	139.25	-544.73	-82.22	113.94
2 sites	4111.19	3241.32	2828.32	278.50	-1033.05	-163.18	249.82
3 sites	4411.38	3181.16	2529.39	417.75	-1472.49	-242.27	409.50
4 sites	4669.59	3121.00	2204.13	557.00	-1869.95	-321.36	595.51
5 sites	4892.69	3058.95	1850.66	696.25	-2232.30	-398.56	809.73
6 sites	5083.84	2996.28	1466.49	835.50	-2562.70	-475.14	1054.65

Pfirmann 4 simulation

$$TR_{P4} = 0.118$$

$TR_{P4WorstCase} = 0.05837;\ TR_{P4BaseCase} = 0.1379;\ TR_{P4BestCase} = 0.3259$

$AST_{P4WorstCase} = 231 \times 0.05837 = 13.483$

$AST_{P4BaseCase} = 231 \times 0.1379 = 31.855$

$AST_{P4BestCase} = 231 \times 0.3259 = 75.283$

$P_{P4\ fen\ extrusion} = P_{non-fen\ extrusion} \times \frac{BST}{AST}$

$P_{P4\ no\ fen\ extrusion} = P_{non-fen\ extrusion} \times TR_{P4} \times BST$

$P_{non-fen\ extrusion} = 0.12$

$P_{P4\ fen\ extrusion,\ worst\ case} = 0.12 \times \frac{231}{13.483} = 2.056$

$P_{P4\ fen\ extrusion,\ base\ case} = 0.12 \times \frac{231}{31.855} = 0.8702$

$P_{P4\ fen\ extrusion,\ best\ case} = 0.12 \times \frac{231}{75.283} = 0.3682$

Consider prophylactic fenestration of 0-6 sites between T11-L4 (T11-T12, T12-T13, T13-L1, L1-L2, L2-L3, L3-L4). Assume non Pfirrmann 4 discs have a baseline chance of extrusion.

$P_{1\leq\ extrusion} = 1 - (1 - P_{P4\ fen\ extrusion,\ X\ case})^{\#\ fenestrated\ discs} \times (1 - P_{non-fen\ extrusion})^{6-\#\ fenestrated\ discs}$

$P_{1\leq extrusion}$ of various cases amongst 0-6 site fenestration					
Sites fenestrated	No fenestration	Worst case	Base case	Best case	Direct cost (£)
	$P_{P4\ no\ fen\ extrusion}$	$P_{P4\ fen\ extrusion}$	$P_{P4\ fen\ extrusion}$	$P_{P4\ fen\ extrusion}$	
0 sites	0.5356	0.5356	0.5356	0.5356	0.00
1 site	1.000	1.000	0.9315	0.6666	139.25

2 sites	1.000	1.000	0.9870	0.7606	278.50
3 sites	1.000	1.000	0.9985	0.8281	417.75
4 sites	1.000	1.000	0.9998	0.8766	557.00
5 sites	1.000	1.000	0.9999	0.9114	696.25
6 sites	1.000	1.000	1.000	0.9364	835.50

$Projected\ costs\ =\ P_{\ 1\leq\ extrusion}\ \times\ £6267.06$

$Total\ costs\ =\ Projected\ costs\ +\ direct\ cost\ of\ fenestration$

$Net\ savings\ =\ 0\ site\ fenestration\ costs\ -\ total\ costs$

Targeted Pfirrmann 4 fenestration cost-benefit analysis								
Sites fenestrated	No fenestration costs	Projected costs worst case	Projected costs base case	Projected costs best case	Direct cost	Net savings worst case	Net savings base case	Net savings best case
0 sites	3356.64	3356.64	3356.64	3356.64	0.00	0.00	0.00	0.00
1 site	6267.06	6267.06	5837.77	4177.62	139.25	-139.25	290.04	1950.19
2 sites	6267.06	6267.06	6185.59	4766.73	278.50	-278.50	-197.03	1221.83
3 sites	6267.06	6267.06	6257.66	5189.75	417.75	-417.75	-408.35	659.56
4 sites	6267.06	6267.06	6265.80	5493.70	557.00	-557.00	-555.74	216.36
5 sites	6267.06	6267.06	6266.43	5711.80	696.25	-696.25	-695.65	-140.99
6 sites	6267.06	6267.06	6267.06	5868.48	835.50	-835.50	-835.50	-436.92

Pfirmann 3 simulation

$TR_{\ P3}\ =\ 0.343$

$$TR_{P3WorstCase} = 0.1697;\ TR_{P3BaseCase} = 0.4010;\ TR_{P3BestCase} = 0.9474$$

$$AST_{P3WorstCase} = 231 \times 0.1697 = 39.201$$

$$AST_{P3BaseCase} = 231 \times 0.4010 = 92.631$$

$$AST_{P3BestCase} = 231 \times 0.9474 = 218.85$$

$$P_{P3\ fen\ extrusion} = P_{non-fen\ extrusion} \times \frac{BST}{AST}$$

$$P_{P3\ no\ fen\ extrusion} = P_{non-fen\ extrusion} \times TR_{P3} \times BST$$

$$P_{non-fen\ extrusion} = 0.12$$

$$P_{P3\ fen\ extrusion,\ worst\ case} = 0.12 \times \frac{231}{39.201} = 0.7071$$

$$P_{P3\ fen\ extrusion,\ base\ case} = 0.12 \times \frac{231}{92.631} = 0.2993$$

$$P_{P3\ fen\ extrusion,\ best\ case} = 0.12 \times \frac{231}{218.85} = 0.1267$$

Consider prophylactic fenestration of 0-6 sites between T11-L4 (T11-T12, T12-T13, T13-L1, L1-L2, L2-L3, L3-L4). Assume non Pfirrmann 3 discs have a baseline chance of extrusion.

$$P_{1\leq\ extrusion} = 1 - (1 - P_{fen\ extrusion,\ X\ case})^{\#\ fenestrated\ discs} \times (1 - P_{non-fen\ extrusion})^{6-\#\ fenestrated\ discs}$$

$P_{1\leq extrusion}$ of various cases amongst 0-6 site fenestration					
Sites fenestrated	No fenestration	Worst case	Base case	Best case	Direct cost (£)
	$P_{P3\ no\ fen\ extrusion}$	$P_{P3\ fen\ extrusion}$	$P_{P3\ fen\ extrusion}$	$P_{P3\ fen\ extrusion}$	
0 sites	0.5356	0.5356	0.5356	0.5356	0.00
1 site	1.000	0.8454	0.6302	0.5391	139.25

2 sites	1.000	0.9486	0.7056	0.5426	278.50
3 sites	1.000	0.9829	0.7656	0.5461	417.75
4 sites	1.000	0.9943	0.8133	0.5496	557.00
5 sites	1.000	0.9981	0.8514	0.5530	696.25
6 sites	1.000	0.9994	0.8816	0.5564	835.50

$$Projected\ costs = P_{1\leq\ extrusion} \times £6267.06$$

$$Total\ costs = Projected\ costs + direct\ cost\ of\ fenestration$$

$$Net\ savings = 0\ site\ fenestration\ costs - total\ costs$$

Targeted Pfirrmann 3 fenestration cost-benefit analysis								
Sites fenestrated	No fenestration costs	Projected costs worst case	Projected costs base case	Projected costs best case	Direct cost	Net savings worst case	Net savings base case	Net savings best case
0 sites	3356.64	3356.64	3356.64	3356.64	0.00	0.00	0.00	0.00
1 site	6267.06	5298.17	3949.50	3378.57	139.25	829.64	2182.31	2749.24
2 sites	6267.06	5944.93	4422.04	3400.51	278.50	43.63	1566.49	2588.05
3 sites	6267.06	6159.89	4798.06	3422.44	417.75	-310.58	1051.25	2426.87
4 sites	6267.06	6231.34	5097.00	3444.38	557.00	-521.28	613.06	2265.68
5 sites	6267.06	6255.15	5335.77	3465.68	696.25	-684.34	235.04	2105.13
6 sites	6267.06	6263.30	5525.04	3486.99	835.50	-831.74	-93.48	1944.57

Sensitivity analysis (Baseline Risk: 0.19)

$$TR_{WorstCase} = 0.4947;\ TR_{BaseCase} = 1.169;\ TR_{BestCase} = 2.762$$

$$AST_{WorstCase} = 231 \times 0.4947 = 114.28$$

$$AST_{BaseCase} = 231 \times 1.169 = 270.04$$

$$AST_{BestCase} = 231 \times 2.762 = 638.02$$

$$P_{fen\ extrusion} = P_{non-fen\ extrusion} \times \frac{BST}{AST}$$

$$P_{non-fen\ extrusion} = 0.03451$$

$$P_{fen\ extrusion,\ worst\ case} = 0.03451 \times \frac{231}{114.28} = 0.06976$$

$$P_{fen\ extrusion,\ base\ case} = 0.03451 \times \frac{231}{270.04} = 0.02952$$

$$P_{fen\ extrusion,\ best\ case} = 0.03451 \times \frac{231}{638.02} = 0.01249$$

Consider prophylactic fenestration of 0-6 sites between T11-L4 (T11-T12, T12-T13, T13-L1, L1-L2, L2-L3, L3-L4).

$$P_{1\leq\ extrusion} = 1 - (1 - P_{fen\ extrusion,\ X\ case})^{\#\ fenestrated\ discs} \times (1 - P_{non-fen\ extrusion})^{6-\#\ fenestrated\ discs}$$

$P_{1\leq extrusion}$ of various cases amongst 0-6 site fenestration				
Sites fenestrated	Worst case	Base case	Best case	Direct cost (£)
0 sites	0.1900	0.1900	0.1900	0.00
1 site	0.2196	0.1858	0.1715	139.25
2 sites	0.2481	0.1816	0.1526	278.50
3 sites	0.2755	0.1774	0.1333	417.75
4 sites	0.3020	0.1731	0.1135	557.00
5 sites	0.3275	0.1688	0.09332	696.25
6 sites	0.3520	0.1646	0.07264	835.50

$$Projected\ costs\ = P_{\ 1\leq\ extrusion} \times £6267.06$$

$$Total\ costs\ = Projected\ costs\ + \ direct\ cost\ of\ fenestration$$

$$Net\ savings\ = 0\ site\ fenestration\ costs\ - \ total\ costs$$

Cost-benefit analysis (baseline risk: 0.19)							
Sites fenestrated	Projected costs worst case	Projected costs base case	Projected costs best case	Direct cost	Net savings worst case	Net savings base case	Net savings best case
0 sites	1190.74	1190.74	1190.74	0.00	0.00	0.00	0.00
1 site	1376.25	1164.42	1074.80	139.25	-324.76	-112.93	-23.31
2 sites	1554.86	1138.10	956.35	278.50	-642.62	-225.86	-44.11
3 sites	1726.58	1111.78	835.40	417.75	-953.59	-338.79	-62.41
4 sites	1892.65	1084.83	711.31	557.00	-1258.91	-451.09	-77.57
5 sites	2052.46	1057.88	584.84	696.25	-1557.97	-563.39	-90.35
6 sites	2206.01	1031.56	455.24	835.50	-1850.86	-676.32	-100.00
